# Anti-Atherogenic Effects of Orlistat on Obesity-Induced Vascular Oxidative Stress Rat Model

**DOI:** 10.3390/antiox10020251

**Published:** 2021-02-06

**Authors:** Zaidatul Akmal Othman, Zaida Zakaria, Joseph Bagi Suleiman, Wan Syaheedah Wan Ghazali, Mahaneem Mohamed

**Affiliations:** 1 Department of Physiology, School of Medical Sciences, Universiti Sains Malaysia, Kubang Kerian, Kelantan 16150, Malaysia; zaidaakmal@unisza.edu.my (Z.A.O.); zaida_zakaria@ymail.com (Z.Z.); bagisuleiman@yahoo.com (J.B.S.); syaheeda@usm.my (W.S.W.G.); 2 Unit of Physiology, Universiti Sultan Zainal Abidin, Kuala Terengganu, Terengganu 20400, Malaysia; 3 Department of Science Laboratory Technology, Akanu Ibiam Federal Polytechnic, Unwana, Ebonyi State P.M.B 1007, Nigeria; 4 Unit of Integrative Medicine, School of Medical Sciences, Universiti Sains Malaysia, Kubang Kerian, Kelantan 16150, Malaysia

**Keywords:** obesity, atherosclerosis, orlistat, antioxidant, anti-inflammatory

## Abstract

Obesity is typically linked to oxidative stress and inflammation, which lead to vascular damage and initiate the progression of atherosclerosis. The aim of this study was to determine the anti-atherosclerotic effect of orlistat on obesity-induced vascular oxidative stress in obese male rats. Twenty-four male Sprague–Dawley rats were categorized into two groups: normal (Normal group, *n* = 6) and high-fat diet (HFD group, *n* = 12). After six weeks, obese rats in the HFD group were administered either with distilled water (OB group) or orlistat 10 mg/kg/day (OB/OR group) for another six weeks. The OB group had a significant increase in lipid profiles (total cholesterol (TC), triglyceride (TG), low-density lipoprotein (LDL)) and decrease in high-density lipoprotein (HDL) level compared to the Normal group. The aortic antioxidants enzymes activities (superoxide dismutase (SOD), glutathione peroxidase (GPx), glutathione reductase (GR), glutathione S-transferase (GST), and catalase (CAT)) as well as total glutathione (GSH) and total antioxidant capacity (TAC) of the OB group were significantly decreased compared to the Normal group. Furthermore, pro-inflammatory atherosclerotic markers (tumour necrosis factor-alpha (TNF-α), vascular cell adhesion molecule-1 (VCAM-1), and intercellular cell adhesion molecule-1 (ICAM-1)) expressions were increased significantly, and anti-inflammatory marker (interleukin-10 (IL-10)) was decreased significantly in the OB group compared to the Normal group. Treatment with orlistat significantly improved lipid profile, increased antioxidant enzymes and expression of anti-inflammatory markers, and decreased the expression of the pro-inflammatory marker compared to the OB group. These findings may suggest the therapeutic effect of orlistat in attenuating the progression of the atherosclerotic stage in obesity.

## 1. Introduction

Obesity has been described as an excessive body fat deposition and may potentially risk the individual health. Global estimation of obesity has reported to constantly increase over the years, including Malaysia, in which approximately 13.10% of adult population were classified as obese [1]. The rise in obesity prevalence has also gained more attention among health professionals in order to prevent various obesity-related diseases and their serious complications such as coronary heart disease, hypertension, diabetes mellitus, metabolic disease, and certain forms of cancer [2]. 

Recent studies have proposed a pathological crosstalk between obesity, oxidative stress, and inflammatory process [3]. Oxidative stress is a phenomenon associated with an imbalance between reactive oxygen species and antioxidant and the failure of biological system to maintain and remove free radical, which further leads to pathological implication. This is further assessed by the presence of advanced oxidation product such as malondialdehyde (MDA) [4]. The oxidative stress and inflammatory progress are co-related and have been shown to mediate the development of atherosclerotic changes in the hyperlipidemic rat model [5]. Atherosclerosis development is exacerbated by the repeated and recurrent mechanism of oxidative alteration and inflammatory processes that are translated into a chronic form [4]. In a condition with excess ingestion of dietary fat, the lipid constituents, particularly the low-density lipoprotein (LDL) cholesterols, are highly permeable to the sub-endothelial layer. The oxidative modification has further taken place and converted the LDL into oxidative form [6]. This consequently surges the rise of MDA formation, which prompts more macrophages to engulf the excess lipids as a part of a clearing process [6]. In response to excess lipids, the presence of macrophages in the sub-endothelial layer is mediated by the major chemokines in the endothelial wall such as the vascular cell adhesion molecule (VCAM-1) and intracellular cell adhesion molecule (ICAM-1) [7]. Orlistat, an anti-obesity pharmacotherapy, has been used as a therapeutic modality and practically prescribed to obese individuals with higher body mass index (BMI) of more than 30 kg/m^2^ and to those who do not meet their goals in lifestyle and dietary interventions [8]. Orlistat has very light side effects on the gastrointestinal tract system, notably diarrhoea, dyspepsia, and flatulence. It has been recognized as one of anti-obesity drugs approved by the Food and Drug Administration (FDA) for long-term usage [9]. Orlistat acts selectively towards gastrointestinal lipase by preventing the hydrolysis of ingested dietary fat into absorbable free fatty acids and glycerol [10]. For this reason, orlistat has demonstrated its effectiveness in improving obesity parameters such as BMI, lipid profiles, white adipocyte size, and fecal fat excretion in animal models, as well as the complications of obesity such as metabolic syndrome and endothelial dysfunction in human [11,12,13]. Considering its easy availability, cost effectiveness, and significant effect on reducing weight, orlistat has become a standard drug and is the most used obesity drug in the wider population with obesity in comparison to other drugs such as sibutramine, which has been shown to cause adverse cardiovascular effects, as well as phentermine and diethylpropion, which have sympathomimetic effects and only subjected for short term management [14]. We previously reported that the supplementation of orlistat with concomitant high-fat diet (HFD) administration for six weeks had demonstrated its protective effects against the increased levels of total cholesterol (TC) and LDL without changes in triglyceride (TG) and high-density lipoprotein (HDL) levels [15]. Additionally, these findings were also associated with absence of atherosclerotic plaque as well as some protective effects against negative changes in cardiac oxidative stress markers and histology [16]. However, to date, there are no reports on the therapeutic effects of oral orlistat on the vascular oxidative stress and inflammatory markers in atherosclerosis of an obese rat model. Therefore, this work was designed to determine the anti-atherogenic effects of orlistat treatment on an obesity-induced vascular oxidative stress rat model, emphasizing its potential anti-oxidant effect on the aorta, which is yet to be explored. The parameters measured were obesity parameters (BMI, Lee index, abdomino-thoracic circumferences), lipid and metabolic profiles (TC, TG, LDL, HDL, leptin and adiponectin), aortic oxidative stress status, and immunoexpression of aortic pro and anti-inflammatory markers, as well as aortic and adipocyte histological changes.

## 2. Materials and Methods

### 2.1. Animal

Eighteen male rats of Sprague–Dawley strain aged between 8–10 weeks weighing 200–250 g were included in this study. The rats were purchased from the laboratory of the Animal Research and Service Centre (ARASC), Universiti Sains Malaysia (USM), Malaysia. Each rat was individually housed in a polypropylene cage, provided with commercial normal pellet (Altromin) by (Altromin Spezialfutter GmbH & Co. KG, Lage, Germany) and drinking water ad libitum during acclimatization period. The animal room was set under controlled temperature at 22–24 °C, relative humidity of 55–70%, and 12-h light/dark cycle.

### 2.2. Preparation of Drug

Orlistat was purchased from Xepa-Soul Pattinson Sdn. Bhd. (Melaka, Malaysia). The dose of orlistat was measured daily according to the individual’s rat weight and dissolved in 1 mL distilled water before being administered to the rats via oral gavage.

### 2.3. Animal Experimental Design

After a one-week acclimatization period, the rats were divided into two groups and fed either with normal pellet (Normal group, *n* = 6/group) or high-fat diet (HFD) (*n* = 12/group) for six weeks to induce obesity. Obese rats were subjected to treatment either with distilled water (1 mL/day) as positive control (OB group, *n* = 6/group) or orlistat at 10 mg/kg/day (OB/OR group, *n* = 6/group) for another six weeks. The dose of orlistat was selected based on a previous study [17]. The rats in the Normal group continued to receive distilled water until the end of experimental period. The guidelines for animal handling were followed according to the National Institute of Health Guide for the Care and Use of Laboratory Animals. The ethic of animal experiment was approved by Institutional Animal Care and Use Committee (IACUC), USM [USM/IACUC/2018/(113)(933)].

### 2.4. Composition of Normal and High-Fat Diet

Altromin pellet (Altromin Spezialfutter GmbH & Co. KG, Lage, Germany) was used to feed the normal control rats and consisted of 24% protein, 12% fat, and 64% carbohydrate. HFD was prepared according to a previous study, which included the combination of Altromin pellet, animal ghee, calcium, vitamin D, and cholesterol powder [15]. This combination has contributed to 12% protein, 31% fat, and 46% carbohydrate. The HFD was shaped into a hand-ball with a small rounded size and prepared freshly a day before being administered to the rats.

### 2.5. Measurement of Body and Nutritional Composition

Body weight of each rat was weighed using digital balance scale (Navigator^TM^, Ohaus Corporation, Nanikon, Switzerland) and recorded on Day 0 and weekly throughout the experimental period. Weight difference was calculated according to the difference between the initial and final body weight. Obesity was described as BMI of more than 0.68 g/cm^2^, according to a previous study [18]. A tape ruler was used to determine the naso-anal length (NAL), body length, and thoracic (immediately behind the foreleg) and abdominal (immediately anterior to the forefoot) circumferences [19]. The administered food was weighed daily (NavigatorTM, Ohaus Corporation, Nanikon, Switzerland) for each individual rat, and the mean of food consumptions was calculated. Total calories was calculated by substitution of food consumption to dietary metabolizable energy.

### 2.6. Blood Sampling and Tissue Preparation

After 12 weeks of the experimental period, rats were fasted overnight and sacrificed under full anaesthesia by intraperitoneal injection of ketamine 90 mg/kg and xylazine 5 mg/kg. The abdominal skin and thoracic cavity were carefully opened, and the a blood sample was immediately collected from posterior vena cava into a serum separator tube. The tube containing collected blood was left for 30 min at room temperature before being centrifuged (Avanti J-HC, Beckman Coulter, IN, USA) at 3000 rpm for 15 min. The supernatant was collected to obtain the serum and was kept at −80 °C until further usage. The aorta was carefully dissected from distal part of abdominal aorta to proximal part of aortic arch. The aorta was removed from adhesive fat and blood and rinsed with normal saline. The proximal aortic arch was weighed and homogenized in phosphate buffer saline solution according to 10% proportion (*w*/*v*). Supernatant was collected and used to measure biochemical profiles. On the other hand, the distal part of the aorta was transversely cut, and ingunal white adipose tissue was harvested and stored in 10% formaldehyde solution for histopathological analysis. The fixed tissues were then processed using automatic tissue processor (Leica TP1020, Nubloch, Baden, Germany) and embedded (Leica, Leider Lane, Lincolnshire, IL, USA) in paraffin wax (Sigma-Aldrich, St. Louis, MO, USA).

### 2.7. Assessment of Lipid, Castelli Risk Indexes, and Metabolic Profiles

TC and TG in serum were determined using commercialized kits (ARCHITECT c kit, Abbott, IL, USA) according to the method of enzymatic colorimetric test. Serum LDL was calculated according to the formula as described in a previous study [20], i.e., LDL (mg/dL) = TC − HDL − (TG/5). Serum HDL was estimated using a commercialized kit (Biosino Bio-Technology and Science Inc, Beijing, China), which involved the removal of chylomicron, LDL-Cholesterol, and VDLD-Cholesterol by cholesterol esterase, cholesterol oxidase, and catalase. Serum leptin and adiponectin were determined by enzyme-link immunoassay kits (Elabscience, Houston, Texas, USA). The Castelli’s risk indexes (CRI) I and II, which indicate the risk of atherosclerosis, were calculated using the formulas as described below:

Castelli Risk Index I (CRI-I) = LDL/HDL [21]

Castelli Risk Index I (CRI-II) = TC/HDL [22]

### 2.8. Fecal Sampling and Determination of Fecal Neutral and Acidic Sterol

At the end of 12th week of the experimental study, the three-day pooled fecal sample of each rat was collected. The amounts of neutral sterol and acidic sterol in the feces were determined using the method described by a previous study [23]. A total of 1 g of feces was pulverized in a mortar and extracted in acetone:ethanol mixture (1:1, *v*/*v*). The extracted feces was diluted in diethyl ether for neutral sterol estimation and in sodium hydroxide solution for acidic sterol estimation. The neutral sterol and acidic sterol were dried and dissolved in acetic acid and continued with the protocol that previously was described, using cholesterol and cholic acid, respectively, as internal standards [24]. The fecal fat excretion was expressed in mg/g.

### 2.9. Assessment of Aortic Oxidative Stress Status

The lipid peroxidation product of malondialdehyde (MDA) level was determined as thiobarbituric acid–reactive substance (TBARS), in which the color complex was produced after the reaction of lipid peroxidation and thiobarbituric acid [25]. The absorbance was read at 532 nm. Total aortic superoxide dismutase (SOD) activity was calculated based on the method described by previous study [26]. The presence of superoxide ion was reduced by tetrazolium blue nitro (NBT) and produced a product of diformazone. The absorbance was read at 560 nm. Catalase (CAT) activity was assayed according to previous study, which involved the decomposition of hydrogen peroxide and its reaction with molybdate ions into yellowish complex form [27]. The absorbance was read at 405 nm. Glutathione peroxidase (GPx) activity was determined by the previous method [28]. The difference in NADPH concentration was read at 340 nm. Glutathione reductase (GR) and glutathione S-transferase (GST) were assayed according to [29] and [30], respectively, and the absorbances were read at 340 nm. Determination of total glutathione (GSH) involved the reaction between sulfhydryl group of GSH with DTNB (5,5’-dithiobis-2-nitrobenzoic acid) reagent [31]. The yellow complex was read at 405 nm. Total antioxidant capacity (TAC) was assayed according to the previous method [32]. This involved the formation of hydroxyl radicals after the reaction of Fe-EDTA complex with hydrogen peroxide. The capacity of antioxidant in the sample to suppress the hydroxyl radicals was determined after the absorbance was read at 532 nm.

### 2.10. Immunohistochemistry

The paraffin embedded thoracic aorta was used to determine the protein expressions of interleukin-10 (IL-10), tumor necrosis factor-alpha (TNF-α), vascular cell adhesion molecule-1 (VCAM-1), and intracellular cell adhesion molecule-1 (ICAM-1). The fixation of aortic tissues in 10% formaldehyde solution was allowed for 48–72 h before being processed (Leica TP1020, Nubloch, Baden, Germany) serially in graded concentrated ethanol. The tissues were embedded (Leica, Leider Lane, Lincolnshire, IL, USA) in paraffin wax (Sigma-Aldrich, St. Louis, MO, USA) and sectioned (Leica, Leider Lane, Wetzlar, Hesse, Germany) to silane-coated slides (Microslides, Muto Pure Chemicals Co., Hongo Bunkyo-ku, Tokyo, Japan) at 5 µm. The sections were deparaffinized twice with xylene and undergone serial rehydration process in decreasing ethanol concentration. The antigen retrieval was achieved by overheating the tissues in pressure cooker at 120 °C containing tris-EDTA, 0.05% Tween buffer, pH 9.0. After washing with distilled water, the tissues were immersed in 3% peroxidase blocking solution for 5 min followed by washing with TBST (Tris Buffer Saline in 0.05% Tween 20, pH 7.4) solution twice, for 5 min per wash. Thereafter, the tissues were incubated with rabbit polyclonal primary antibody of TNF-α (Cloud Clone, 1:120), IL-10 (Cloud Clone, 1:100), VCAM-1 (Cloud Clone, 1:60), and ICAM-1 (Cloud Clone, 1:100) overnight in a humidified container at 4 °C. Following that, the tissues were washed with TBST twice for 5 min each and incubated with goat, anti-rabbit secondary antibody containing HRP conjugate (Dako North America, Inc., CA, USA) for 1 h. Diaminobenzidine (Dako North America, Inc., CA, USA) was applied as chromogen substrate and the slides were briefly counterstained with Harris hematoxylin (Merck, Darmstadt, Germany). The expressions of IL-10, TNF-α, VCAM-1, and ICAM-1 were observed in endothelial and vascular layers, and the images were captured (Olympus BX41, Tokyo, Japan) at 1000× magnification, which connected to Cellsense imaging software. Image J Software (ImageJ, NIH-Bethesda, MD, USA) was used as a tool to calculate the percentage area and intensity of five non-overlapped images of each rat (*n* = 6/group).

### 2.11. Hematoxylin and Eosin Staining of Aorta and White Adipose Tissue

The paraffin-embedded aortic and adipose tissues were deparaffinized twice in xylene and rehydrated in ethanol before being sectioned (Leica, Leider Lane, Wetzlar, Hesse, Germany) at 5 µm thick. Thereafter, the aortic and adipose tissue sections were stained with hematoxylin and eosin (H&E) (Merck, Frankfurter, Darmstadt, Germany). All slides were mounted with Cytoseal mounting medium (Richard-Allan Scientific™, Kalamazo, MI, USA). The images were obtained by light microscope (Olympus BX41, Shinjuku-ku, Tokyo, Japan) at 1000× magnification for the aortic section to visualize the endothelial surface and alignment of aortic elastic lamina, and at 400× magnification for the adipose tissue section to assess the adipocyte size. Image J Software (ImageJ, NIH-Bethesda, Maryland, USA) was used to analyze the surface area, perimeter, and number of adipocyte in the adipose tissue.

### 2.12. Toluidine Blue Staining of Aorta

The paraffin-embedded aortic sections were deparaffinized twice in xylene, rehydrated in serial decreasing of ethanol concentrations, and sectioned (Leica, Leider Lane, Wetzlar, Hesse, Germany) at 5 µm thick before being immersed in 1% aqueous toluidine blue solution for 5 min. The sections were rinsed in distilled water, dehydrated twice in acetone, cleared twice with xylene, and mounted with Cytoseal (Richard-Allan Scientific™, Kalamazo, MI, USA). The sections were visualized under a light microscope (Olympus BX41, Shinjuku-ku, Tokyo, Japan) at 1000× magnification to assess the presence of monocyte near endothelial layer.

### 2.13. Statistical Analysis

Statistical analysis was conducted using GraphPad Prism, 8th Version Software (GraphPad Software Inc., Maryland, USA). All data were checked for normality and variance of the data sets by using Shapiro–Wilk and D’Agostino–Pearson Omnibus normality tests, respectively. The statistical differences among the means were determined using one-way analysis of variance (ANOVA) followed by the Tukey method for post-hoc analysis. The results were expressed as mean and standard error of the mean (SEM). *P* value of less than 0.05 was regarded as statistically significant.

## 3. Results

### 3.1. Effect of Orlistat on Body and Nutritional Composition

**Figure 1:**
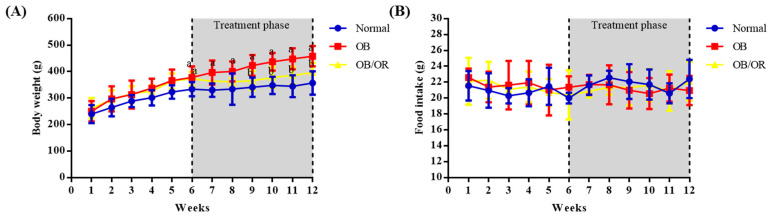
Effects of orlistat on (**A**) body weight and (**B**) food intake in all experimental groups. Treatment with orlistat (10 mg/kg/day) was started after six weeks of obesity induction. Each bar represents mean (with standard error of mean), *n* = 6 per group. OB; Obese, OB/OR; Obese + orlistat 10 mg/kg/day. a = *p* < 0.05 OB group compared to Normal group; b = *p* < 0.05 OB/OR group compared to OB group.

The trend of body weight was observed to be increased in all groups along the experimental period (Figure 1A). After six weeks of obesity induction, HFD significantly increased the body weight in OB and OB/OR groups compared to Normal group. From then on, the body weight of rats in the OB group continued to be significantly increased throughout the experimental period. Orlistat treatment persistently restored the increased body weight, which was significantly observed at the ninth week until the end of the experimental period (Figure 1A). No significant difference of food intake was observed between all groups at each week of experimental period, which indicated that the total food intake was similar in all groups (Figure 1B).

Table 1 shows the difference in body and nutritional composition of all groups after 12 weeks of the experimental period. The initial body weight did not significantly differ between all groups at the start of the animal experiment. After 12 weeks of the experimental period, significant increases were observed in final body weight, weight difference, BMI, TC, AC, as well as AC/TC ratio of the OB group as compared to the Normal group. Differently, rats in the OB/OR group had shown significant decreases in these obesity markers as compared to the OB group. The mean food intakes were similar in all experimental groups. However, rats in the OB and OB/OR groups had a significant higher calorie intake in comparison to the Normal group.

### 3.2. Effect of Orlistat on Lipid and Metabolic Profiles

The assessment of serum lipid and metabolic profiles were carried out for all groups. There were significant increases for the levels of TC, TG, LDL, CRI I and II, and leptin but significant decrease for the levels of HDL and adiponectin in the OB group as compared to the Normal group. Daily oral administration of orlistat in the obese rats in concomitant to HFD for six weeks significantly decreased the levels of TC, TG, LDL, CRI-I, CRI-II, and leptin but significantly increased the levels of HDL and adiponectin compared to the OB group (Table 2).

### 3.3. Effects of Orlistat on Fecal Neutral and Acidic Sterol

The levels of fecal neutral and acidic as well as total fecal sterol were significantly increased in the OB group as compared to the Normal group. Meanwhile, both fecal neutral and acidic sterols as well as total fecal sterol were significantly increased in the OB/OR group compared to the Normal and OB groups (Table 3).

### 3.4. Effect of Orlistat on Changes in Aortic Oxidant/Antioxidant Markers

To determine the effects of orlistat therapy on vascular oxidative stress markers, we determined the levels of oxidant and antioxidant markers, which include MDA as oxidative stress marker, antioxidant enzymatic activities of SOD, CAT, GPx, GR, and GST, as well as GSH, and TAC levels. It was demonstrated that the level of aortic MDA was significantly increased, while the activities of SOD, CAT, GPx, GR, and GST as well as GSH and TAC levels were significantly decreased in the OB group as compared to the Normal group. Daily administration of orlistat in the obese rats for six weeks, with concomitant HFD administration, had significantly reversed these changes (Table 4).

### 3.5. Effect of Orlistat on Changes in Aortic Inflammatory Markers

**Figure 2:**
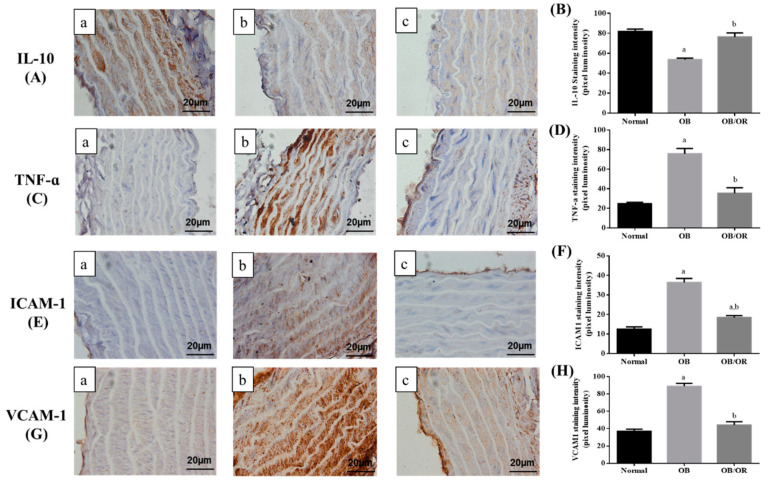
Expressions of IL-10 (**A**,**B**), TNF-α (**C**,**D**), ICAM-1 (**E**,**F**), and VCAM-1 (**G**,**H**) in the aorta of (a) Normal, (b) OB, and (c) OB/OR groups (magnification = 1000×, scale bar = 20 µm). For quantitative data, values are expressed as means and standard error of means, *n* = 6. ^a^ *p* < 0.05 compared with Normal group; ^b^ *p* < 0.05 compared with OB group (one-way ANOVA followed by Tukey’s post-hoc test). OB: obese, OB/OR: obese and orlistat 10 mg/kg/day, IL-10: interleukin-10, TNF-α: tumour necrosis factor alpha, ICAM-1: intracellular adhesion molecule 1, VCAM-1: vascular cell adhesion molecule 1.

To assess the effect of orlistat therapy on the changes in the inflammatory markers, we performed the expressions of selected anti- and pro-inflammatory proteins such as IL-10, TNF-α, VCAM-1, and ICAM-1. Our study had shown a significant decrease in the expression of IL-10 (Figure 2A), with significant increases of TNF-α (Figure 2C), ICAM-1 (Figure 2E), VCAM-1 (Figure 2G) and expressions in the aorta of the OB group as compared to the Normal group. In addition, these markers were significantly improved following orlistat intervention. Results from quantitative data analysis study had shown a significant decrease in the intensity of IL-10 immunoexpression (Figure 2B) in the OB group relative to the Normal group, with a significant increase in the intensity of IL-10 immunoexpression (Figure 2B) of the OB/OR group, relative to the OB group. The intensity of TNF-α (Figure 2D), ICAM-1 (Figure 2F), and VCAM-1 (Figure 2H) were significantly increased in the OB group relative to the Normal group, and the intensity of these markers were found to be significantly decreased in the OB/OR group relative to the OB group.

### 3.6. Effects of Orlistat on Histopathological Changes

**Figure 3:**
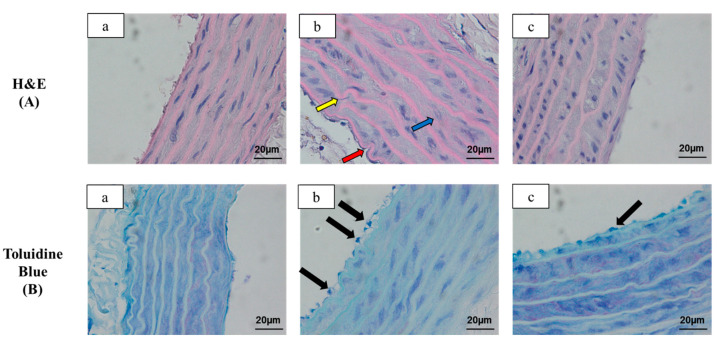
Representative images of transverse sections of thoracic aorta under (**A**) H&E and (**B**) toluidine blue staining in (**a**) Normal, (**b**) OB, and (**c**) OB/OR groups (magnification = X1000, scale bar = 20 µm). For H&E staining, the endothelial surface of OB group appeared to be rough (red arrow) with the presence of tortuous elastic lamina (yellow arrow) and thickening of interlamina space (blue arrow) compared to the Normal group. The pathological effects of aorta were improved in OB/OR group as evidence of flattened endothelial surface and less thickened interlamina space compared to the OB group. Furthermore, the internal lamina lining was improved without the presence of tortuous fibres. For toluidine blue staining, there was copious adhesion of mononuclear cells near to endothelial surface (Black arrow) in the OB group compared to the Normal and OB/OR groups. OB: obese, OB/OR: obese and orlistat 10 mg/kg/day, H&E: hematoxylin and eosin staining.

**Figure 4:**
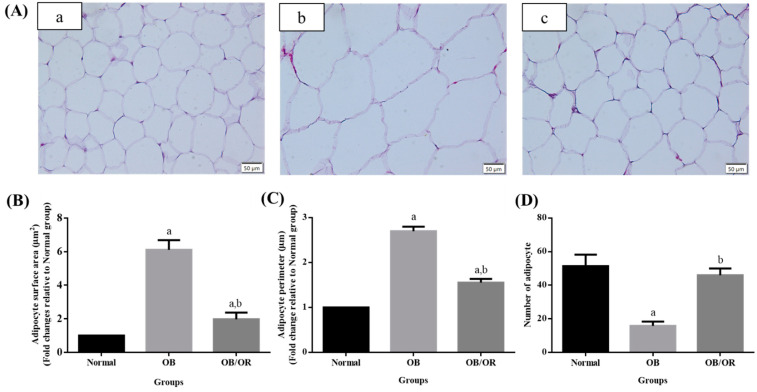
Pictomicrograph of white adipose tissues in (**A**) H&E staining (magnification = X400, scale bar = 50 µm) and histomorphometric analysis of (**B**) adipocyte area, (**C**) adipocyte perimeter, and (**D**) number of adipocyte in (a) Normal, (b) OB, and (c) OB/OR groups. The adipocyte surface area and perimeter were normalized to the value of the Normal group. The results were presented as mean and S.E.M (*n* = 5 sections per animal). ^a^ *p* < 0.05 compared with the Normal group; ^b^ *p* < 0.05 compared with the OB group (one-way ANOVA followed by Tukey’s post-hoc test). OB: obese, OB/OR: obese and orlistat 10 mg/kg/day, H&E: hematoxylin and eosin.

Elastic lamina was assessed by H&E staining (Figure 3A), while the presence of adhered mononuclear cells to endothelium surface was determined by toluidine blue staining (Figure 3B). From the findings of the H&E staining, the aorta of the Normal group had was intact, which demonstrated the presence of smooth endothelial surface containing flat monolayer endothelial cells and the intima containing their matrix. The media layer showed the presence of internal elastic lamina that homogeneously arranged. In contrary, the aortic wall of the OB group was obviously thicker, and the endothelial surface appeared to be rough when compared to the Normal group. The elastic lamina space was widened and irregularly arranged with the presence of tortuous fibres. These pathological changes were alleviated in the OB/OR group as evidence of flattened endothelial surface with reduced in thickening of interlamina space when compared to the OB group. There was also some irregularity of internal lamina without the presence of tortuous fibres. Based on our findings in aortic toluidine blue staining, the aorta of the OB group showed copious adhesion of mononuclear cells at the endothelial surface. In contrast, the presence of mononuclear cell was shown to be markedly reduced in the OB/OR group when compared to the OB group. Figure 4 represented the image of adipocyte in H&E staining from each experimental group (Figure 4A). Based on our findings, the adipocyte size of the Normal group was in signet ring appearance with the presence of thin cytoplasm and small rounded nucleus. Significant increases in surface area and perimeter of adipocytes were observed in OB and OB/OR groups, indicating that the administration of HFD for 12 weeks caused hypertrophy of the adipocytes when compared to the Normal group (Figure 4B,C). The adipocyte number was significantly reduced in the OB group compared to Normal group and significantly increased in the OB/OR group when compared to OB group (Figure 4D).

## 4. Discussion

Obesity is independently associated with higher risk for cardiovascular disease (CVD), particularly atherosclerosis [33]. Obesity has a significant effect on the increase of coronary risk in the presence of associated metabolic and vascular disturbances such as dyslipidemia, hypertension, insulin resistance, and inflammation [34]. High consumption of fatty diet is one of the major factors in developing obesity. In our previous study, we demonstrated that the administration of orlistat at 10 mg/kg/day concomitantly with HFD for six weeks had shown a protective effect against the increased Lee obesity index, decreased cardiac antioxidants levels, and increased lipid peroxide derivative of MDA level in rats [16]. In the present study, the rats were given HFD for six weeks to establish an obese condition. Then the obese rats were treated with orlistat at 10 mg/kg/day orally in combination with HFD for another six weeks. We aimed to determine the potential anti-atherogenic effects of orlistat treatment on obesity-induced vascular oxidative stress in an obese rat model. Atherosclerosis encompasses a condition whereby there is an aggregation of different classes of cells such as monocytes, T-helper cells, and platelets, which are present in response to inflammation [35]. This is aided by the presence of chemokines such as TNF-α and activation of abundance of adhesion molecules such as VCAM-1 and ICAM-1 [8]. In addition, the oxidative stress caused by the excess lipid in circulation has become an accelerated factor in early stage of atherosclerosis [36].

In the present study, HFD consists of five major components, which include animal ghee, cholesterol powder, powdered chow pellet, vitamin D, and calcium, and these corresponded to higher fat composition of up to 31% compared to the normal diet, which accounted for 12%. Following a 12-week continuous HFD administration in the OB group, our results demonstrated significant increase in obesity parameters, which included BMI value, body weight difference, TC, AC, and AC/TC ratio relative to the Normal group. This might be due to the higher fat composition in our HFD regime, which further increased the build-up of fat mass in the abdominal and visceral regions [37]. Our finding was also consistent with a study that established an obese rat model that used HFD comprised of 58% fat content for 10 weeks, and consequently caused increased body weight gain and body fat mass [38]. This was explained by the principal of dietary fat contributing body mass, although the experimental period and diet fat regime were longer and higher, respectively. In addition, the excess calories also caused an excess energy density, which in turn increases the body weight gain [39]. The increase in the obesity parameters observed in our findings might also be attributed to the excessive accumulation of adipose tissue mass, as shown by the increase in adipocyte size in the OB group. Our study has demonstrated the presence of increased adipocyte size in the OB group, which could be explained by the excess storage of fat from the HFD leading to the significant higher body weight gain compared to the Normal group. The increase in adiposity has been associated with increase in macrophage infiltration, extracellular matrix remodelling, and inflammatory response in the adipose tissue of an obese animal model [40], which is suggested to be assessed in a future study together with its adipokine level. This might be a crucial pathological link for the development of obesity-related complications. In contrast, the significant lower body weight gain in the OB/OR group could be attributed to the presence of its smaller adipocyte size when compared to the OB group. On the other hand, significant decreases of the obesity parameters were observed in the OB/OR group in comparison to the OB group, showing that orlistat is a potent gastro-pancreatic lipase drug that is capable of reducing the body weight after six weeks of the therapeutic period. These results have also strongly supported the evidence that orlistat has a decrement effect on the percentage of absorbable dietary fat at a maximum of 30% [41].

Obesity-related vascular disorders are generally associated with increased levels of TC, TG, and oxidized LDL [5]. Similarly, our study revealed that the levels of TC, TG, and LDL were significantly increased in the OB group compared to the Normal group. High levels of these lipid profiles, particularly LDL cholesterol, which have a pro-atherogenic property can cause damages to the cellular and macromolecule contents [42]. Orlistat has shown its significant decreasing effect towards these lipid profile levels in the OB/OR group when compared to the OB group. This finding indicates that besides reducing the body weight, orlistat has also been shown to alter the levels of isolated lipid fractions as a result from its inhibitory effect on pancreatic lipase activity [12], which in turn decreases the free fatty acids absorbance at the intestinal level. The orlistat binds covalently to serine residue of active site on lipase enzyme and reduces its activity [43]. These findings indicate that orlistat also has its contributing effect in alleviating the higher lipid level. This was further supported by our findings on the significantly higher fecal fat content in the OB/OR group. The increased fecal acidic and sterol contents may also contribute to the concomitant finding on the smaller adipocyte size found in this group.

Castelli risk indexes were used to predict the atherosclerosis risk by calculating the ratio of isolated lipid fractions [22]. Our findings showed that the risk of atherosclerosis was significantly greater in the OB group as compared to the Normal group. Meanwhile, the atherosclerosis risk was significantly reduced after administration of orlistat for six weeks in the obese rats. The decrease of HDL anti-atherogenic cholesterol in the OB group may predispose the vessel to atherogenesis, which can explain the interplay between obesity, dyslipidemia, and atherosclerosis. We observed a significant increase of HDL level in the OB/OR group compared to the OB group consistent with previous studies [12,44], suggesting the lower atherosclerosis risk found in this group. The similar finding on the increased HDL level is also found in humans, as reported by a previous study [45]. However, another human study has also shown insignificant difference on HDL level [46]. These discrepancies could be due to the difference in the type of diet intake during the studies and the difference between species [47]. Hence, orlistat might be useful as a co-treatment to the obese model to combat obesity and dyslipidemia, which are co-related.

Obesity is a state of chronic oxidative stress manifested by the increase in reactive oxygen species (ROS) and impairment in antioxidant defence. ROS initiates vascular damage by causing alteration in vascular tone and vascular inflammation [48]. An increase in MDA level is an important indicator for the presence of oxidative stress due to the process of lipid peroxidation [49]. MDA is one of the reactive species and has been an objective focus for therapeutic targets in many pharmaceutical drugs in treating atherosclerosis. In our study, the increased aortic MDA level along with the decreased SOD, CAT, GPx, GST, and GR activities, as well as decreased TAC and GSH levels in the OB group, suggests the presence of oxidative stress, which can accelerate the progress of atherosclerosis. On the other hand, our findings showed that orlistat treatment had significantly improved the aortic oxidative stress status in obese male rats, which is similar to previous studies [50,51,52]. The improvement in the oxidative stress level might be attributed to the ability of orlistat in preventing pro-atherogenic LDL cholesterol from oxidation. On the other hand, the increase in HDL found in the OB/OR group not only protects the progress of atherosclerosis by reverse cholesterol transport, but also by its action on hydrolyzing lipid peroxidation product. Orlistat has demonstrated to increase paraoxonase (PON1) activity of HDL that is responsible for exhibiting the antioxidant effect of HDL [53]. This finding also might explain the decreased level of MDA found in OB/OR group in our present study. Additionally, a study has also shown that the hyperlipidemia state impairs the antioxidant defence capacity and raises lipid peroxidation products [54]. Obesity is a known risk factor of oxidative stress and atherosclerosis. Therefore, the improved oxidative stress markers might also be directly attributed, which could be demonstrated in future study.

Several lines of evidence have indicated that obesity-related vascular dysfunction is not only contributed to by hyperlipidemic condition, but also by the inflammatory process. VCAM-1 and ICAM-1 are the chemokines that functionally act by adhering the monocyte into subendothelial space, which are found to be numerous in the endothelium at proinflammatory state [55]. In the condition of higher inflammatory state, the invaded macrophages tend to secrete inflammatory cytokine such as TNF-α and further trigger the inflammatory cascade by activating the foam cells formation [56]. The previous study has shown that TNF-α is a key pro-inflammatory marker, which also upregulates the VCAM-1 and ICAM-1 expressions and stimulates the adherence of monocytes to the endothelial surface [57]. The TNF-α is primarily secreted by macrophages and further triggers the release of more inflammatory cytokines such as IL-1β, IFN-γ, and IL-6 [58]. We observed significant increases in the expressions of pro-inflammatory marker of TNF-α and adhesion molecules VCAM-1 and ICAM-1, with simultaneous decrease in the expression of anti-inflammatory marker of IL-10 in the OB group. These findings were in an agreement with our histological findings, which showed the adherence of monocytes onto the endothelial surface of the aorta stained with toluidine blue in the OB group. The migrated monocytes can further trigger the cascades of inflammatory process and aggravate the atherosclerosis process. The infiltration of the inflammatory cells and hyperplasia of the fibrous matrix in the sub-endothelial area can lead to a thicker and wider vascular wall [59], as observed in the OB/OR group, in which the aorta wall was thicker with the presence of tortuous elastic lamina in comparison to the Normal group.

We also demonstrated for the first time that there were significant downregulations of pro-inflammatory markers TNF-α, VCAM-1, and ICAM-1 and significant upregulation of anti-inflammatory marker IL-10 in the aorta of the OB/OR group relative to the OB group, which could be due to the decreased aortic MDA level. This is further supported by the presence of less tortuous elastic lamina of the aorta observed in the OB/OR group when compared to the OB group. These findings may indicate that orlistat treatment for six weeks in obese rats can reduce monocyte adhesion to the endothelial surface of the aorta suggesting the anti-atherogenic role of orlistat in the atherosclerosis progression.

Apart from the roles of antioxidants and anti-inflammatory pathways in reducing the atherogenic process, adiponectin has also been targeted as the main role that interplays between these atherogenesis factors. Adiponectin, an anti-inflammatory adipocytokines, is primarily dispensed by adipose tissue, and it is declined in obesity. The biological function of adiponectin has been studied over the last decade, and the majority of studies have shown that it has a major influence on lipid metabolism and a major interplay for atheropathogenesis [60]. Previous experimental data indicated that adiponectin was able to suppress the expressions of TNF-α and its downstream inflammatory cytokines [50]. Our present study demonstrated that the adiponectin level in the OB group was significantly decreased in comparison to the Normal group, which was parallel to other study [61]. This could be due to the higher inflammatory cytokines in the OB group, which could further suppress the adiponectin level [11]. In addition, adiponectin and leptin are both co-related towards the progression of atherosclerosis [62]. The leptin level in our study was significantly increased in the OB group compared to the Normal group. This higher leptin level was correlated to higher macrophage infiltration due to its effect as a macrophage recruiter, which further increases the rate of atherosclerosis progression [63]. After six weeks of treatment with orlistat in the obese male rats, the level of leptin was significantly decreased, and the level of adiponectin was significantly increased compared to the OB group. The improvement of leptin and adiponectin levels in the OB/OR group was possibly due to its smaller adipose tissue compared to OB group.

## 5. Conclusions

In conclusion, orlistat treatment at 10 mg/kg/day for six weeks significantly mitigated the increased aortic oxidative stress and inflammation, partly via its effect in improving lipid profile, which in turn may attenuate the progression of atherosclerosis in obese rats. Therefore, orlistat may possess a therapeutic effect in attenuating the progression of atherosclerosis in obesity. However, future study is recommended to investigate its exact molecular mechanism. In addition, it is also suggested to study the effect of orlistat on oxidative stress and inflammation in adipose tissue to further support this conclusion.

## Figures and Tables

**Table 1:** Effects of orlistat on body and nutritional composition.

	**Normal**	**OB**	**OB/OR**
Initial body weight (g)	249.80 (8.51)	251.10 (11.70)	254.40 (13.96)
Final body weight (g)	358.10 (12.40)	474.00 (12.12) ^a^	396.60 (14.94) ^b^
Weight difference (g)	112.20 (8.53)	202.40 (11.80) ^a^	161.70 (10.23) ^a,b^
BMI	0.67 (0.01)	0.82 (0.02) ^a^	0.74 (0.02) ^a,b^
TC (cm)	15.50 (0.24)	17.00 (0.25) ^a^	16.18 (0.28)
AC (cm)	16.38 (0.31)	18.04 (0.35) ^a^	16.69 (0.41) ^b^
AC/TC ratio	1.04 (0.01)	1.14 (0.01) ^a^	1.07 (0.02) ^b^
Food intake (g/day)	21.28 (0.45)	20.84 (0.74)	20.83 (0.83)
Calorie intake (kcal/day)	70.07 (1.63)	102.80 (3.28) ^a^	103.60 (4.01) ^a^

Data are presented as means (with standard error of means), *n* = 6 per group. OB: Obese, OB/OR: Obese + orlistat 10 mg/kg/day, BMI: body mass index, TC: thoracic circumference; AC: abdominal circumference. ^a^ *p* < 0.05 compared with Normal group; ^b^ *p* < 0.05 compared with OB group (one-way ANOVA followed by Tukey’s post-hoc test).

**Table 2:** Effect of orlistat on lipid and metabolic parameters.

	**Normal**	**OB**	**OB/OR**
TC (mg/dL)	60.79 (2.28)	83.89 (3.98) ^a^	73.73 (3.09) ^b^
TG (mg/dL)	50.54 (3.11)	78.98 (3.35) ^a^	62.75 (6.11) ^b^
LDL (mg/dL)	34.25 (2.45)	59.94 (5.35) ^a^	41.57 (3.25) ^b^
HDL (mg/dL)	19.72 (0.69)	14.92 (0.55) ^a^	22.88 (1.46) ^b^
CRI-1	1.85 (0.15)	3.53 (0.26) ^a^	2.04 (0.21) ^b^
CRI-11	3.43 (0.19)	5.05 (0.31) ^a^	3.60 (0.22) ^b^
Leptin (ng/mL)	1.51 (0.11)	7.42 (0.22) ^a^	3.19 (0.14) ^b^
Adiponectin (ng/mL)	3.39 (0.24)	2.24 (0.11) ^a^	3.25 (0.07) ^b^

Data are presented as means (with standard error of means), *n* = 6 per group. OB: Obese, OB/OR: Obese + orlistat 10 mg/kg/day, TC: total cholesterol, TG: triglyceride, LDL: low-density lipoprotein, HDL: high-density lipoprotein, CRI: Castelli’s risk index. ^a^ *p* < 0.05 compared with Normal group; ^b^ *p* < 0.05 compared with OB group (one-way ANOVA followed by Tukey’s post-hoc test).

**Table 3:** Fecal neutral and acidic sterol.

	**Normal**	**OB**	**OB/OR**
Neutral sterol (mg/g)	6.42 (0.63)	54.23 (0.25) ^a^	59.27 (0.81) ^a,b^
Acidic sterol (mg/g)	10.75 (0.94)	93.98 (1.46) ^a^	102.10 (0.92) ^a,b^
Total fecal sterol (mg/g)	18.06 (3.89)	149.60 (2.51) ^a^	160.90 (4.49) ^a,b^

Data are presented as means (with standard error of means), *n* = 6 per group. OB: Obese, OB/OR: Obese + orlistat 10 mg/kg/day. ^a^ *p* < 0.05 compared with Normal group; ^b^ *p* < 0.05 compared with OB group (one-way ANOVA followed by Tukey’s post-hoc test).

**Table 4:** Effect of orlistat on vascular oxidative stress markers.

	**Normal**	**OB**	**OB/OR**
MDA level (nmol/mg protein)	2.02 (0.27)	10.00 (0.86) ^a^	2.72 (0.62) ^b^
SOD activity (unit/mg protein)	137.90 (7.44)	54.30 (7.39) ^a^	115.50 (10.54) ^b^
CAT activity (unit/mg protein)	107.60 (9.34)	57.32 (8.20) ^a^	97.30 (5.94) ^b^
GPx activity (unit/mg protein)	1.85 (0.15)	0.54 (0.08) ^a^	1.39 (0.27) ^b^
GST activity (unit/mg protein)	11.81 (0.86)	6.89 (1.49) ^a^	11.26 (0.94) ^b^
GR activity (unit/mg protein)	1.62 (0.12)	0.86 (0.13) ^a^	1.40 (0.12) ^b^
GSH level (nmol GSH Eq./mg protein)	16.83 (1.00)	10.91 (0.58) ^a^	13.52 (0.40) ^a,b^
TAC level (nmol uric acid Eq./mg protein)	233.00 (7.18)	174.80 (8.98) ^a^	220.10 (9.03) ^b^

Data are presented as means (with standard error of means), *n* = 6 per group. OB: Obese, OB/OR: Obese + orlistat 10 mg/kg/day, MDA: malondialdehyde, SOD: superoxide dismutase, CAT: catalase, GPx: glutathione peroxidase, GST: glutathione S-transferase, GR: glutathione reductase, GSH: glutathione, TAC: total antioxidant capacity. ^a^ *p* < 0.05 compared with Normal group; ^b^ *p* < 0.05 compared with OB group (one-way ANOVA followed by Tukey’s post-hoc test).

## Data Availability

The data are presented within the paper. Additional raw data are available on request from the corresponding author.
